# 2-*r*-(4-Chloro­phen­yl)-6-*c*-phenyl-3,4,5,6-tetra­hydro-2*H*-thio­pyran-4-one 1-oxide

**DOI:** 10.1107/S1600536808037355

**Published:** 2008-11-20

**Authors:** A. Thiruvalluvar, S. Balamurugan, R. J. Butcher, K. Pandiarajan, D. Devanathan

**Affiliations:** a PG Research Department of Physics, Rajah Serfoji Government College (Autonomous), Thanjavur 613 005, Tamilnadu, India; b Department of Chemistry, Howard University, 525 College Street NW, Washington, DC 20059, USA; c Department of Chemistry, Annamalai University, Annamalai Nagar 608 002, Tamilnadu, India

## Abstract

The thio­pyran unit of the title mol­ecule, C_17_H_15_ClO_2_S, is in chair form. A crystallographic mirror plane bis­ects the mol­ecule, passing through the O=S and the opposite C=O atoms of the central ring, with statistical disorder of the Cl atom. The geometry around the S atom is tetra­hedral and the carbonyl C is planar. The 4-chloro­phenyl group at the 2 position and the phenyl ring at the 6 position have equatorial orientations. Inter­molecular C—H⋯O and C—H⋯Cl hydrogen bonds are found in the crystal structure. In addition, there is a short O⋯C inter­molecular contact [2.970 (5) Å].

## Related literature

For a related crystal structure, see: Thiruvalluvar *et al.* (2007 ▶). For applications of sulfoxides, see: Contreras *et al.* (1998 ▶); Hutton *et al.* (2002 ▶); Okada & Tanaka (2002 ▶). For the conformational analysis of substituted thian-1-oxides, see: Freeman *et al.* (2001 ▶); Nagao *et al.* (1995 ▶). For the anti­microbial activity of aliphatic, aromatic and cyclic sulfoxides, see: Ansel *et al.* (2006 ▶); Ingold *et al.* (1999 ▶); Rouvier *et al.* (2004 ▶).
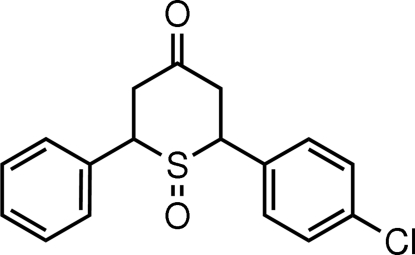


## Experimental

### 

#### Crystal data

C_17_H_15_ClO_2_S
*M* *_r_* = 318.81
Orthorhombic, 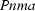

*a* = 11.5195 (5) Å
*b* = 25.7589 (12) Å
*c* = 5.2248 (2) Å
*V* = 1550.35 (12) Å^3^
*Z* = 4
Mo *K*α radiation
μ = 0.38 mm^−1^
*T* = 296 (2) K
0.58 × 0.35 × 0.15 mm

#### Data collection

Bruker APEXII CCD diffractometer
Absorption correction: multi-scan (*SADABS*; Bruker, 2004 ▶) *T*_min_ = 0.809, *T*_max_ = 0.945
33450 measured reflections
1954 independent reflections
1551 reflections with *I* > 2σ(*I*)
*R*_int_ = 0.040

#### Refinement

*R*[*F* ^2^ > 2σ(*F* ^2^)] = 0.060
*wR*(*F* ^2^) = 0.211
*S* = 1.20
1954 reflections
106 parameters
H-atom parameters constrained
Δρ_max_ = 0.32 e Å^−3^
Δρ_min_ = −0.42 e Å^−3^

### 

Data collection: *APEX2* (Bruker, 2004 ▶); cell refinement: *APEX2*; data reduction: *SAINT-NT* (Bruker, 2004 ▶); program(s) used to solve structure: *SHELXS97* (Sheldrick, 2008 ▶); program(s) used to refine structure: *SHELXL97* (Sheldrick, 2008 ▶); molecular graphics: *ORTEP-3* (Farrugia, 1997 ▶); software used to prepare material for publication: *PLATON* (Spek, 2003 ▶).

## Supplementary Material

Crystal structure: contains datablocks global, I. DOI: 10.1107/S1600536808037355/hg2443sup1.cif

Structure factors: contains datablocks I. DOI: 10.1107/S1600536808037355/hg2443Isup2.hkl

Additional supplementary materials:  crystallographic information; 3D view; checkCIF report

## Figures and Tables

**Table 1:** Hydrogen-bond geometry (Å, °)

*D*—H⋯*A*	*D*—H	H⋯*A*	*D*⋯*A*	*D*—H⋯*A*
C2—H2⋯O1^i^	0.98	2.43	3.232 (4)	138
C15—H15⋯Cl1^ii^	0.93	2.82	3.745 (9)	170

Symmetry codes: (i) 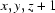; (ii) 
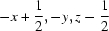
.

**Fig. 1.**
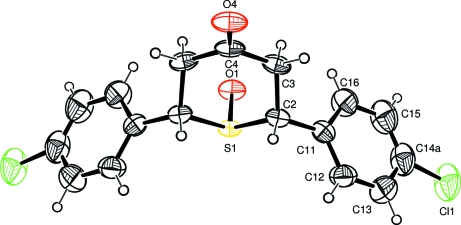
The molecular structure of the title compound with the atomic numbering and 30% probability displacement ellipsoids. The unlabelled and labelled atoms are related by mirror plane [symmetry code: x, 1/2 - y, z].

**Fig. 2.**
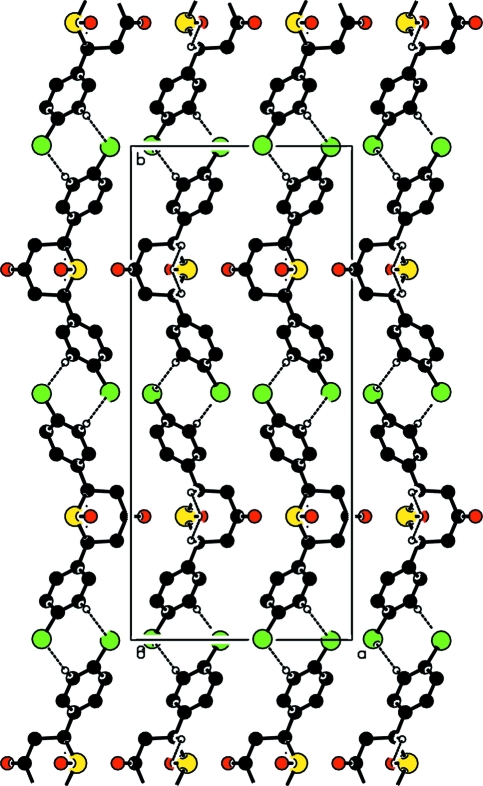
The packing of the title compound, viewed down the c axis. Dashed lines indicate hydrogen bonds. H atoms not involved in hydrogen bonding have been omitted.

## supplementary crystallographic information

### Comment

Increasing interest has been focused on the stereochemical aspects of sulphoxides. The conformational analysis of cyclic sulphoxides is an area of attraction for many groups (Okada & Tanaka, 2002; Hutton *et al.*, 2002; Contreras *et al.*, 1998). Significant attention was earlier directed toward the conformational analysis of substituted thian-1-oxides (Freeman *et al.*, 2001; Nagao *et al.*, 1995). The conformational integrity of the sulphoxide has also generated interest in the various conditions under which it may undergo stereoisomerization. A large number of aliphatic, aromatic and cyclic sulphoxides exhibit antimicrobial activity (Ingold *et al.*, 1999; Ansel *et al.*, 2006; Rouvier *et al.*, 2004). Although extensive studies on sulphoxides have disclosed their chemical and physico-chemical properties, little is known about the properties of the sulphinyl groups in cyclic sulphoxides. To investigate the conformations, substitution effect and antimicrobial activity of unsymmetrical thiopyran-4-one 1-oxides, the title compound was synthesized.

Thiruvalluvar *et al.*, (2007) have reported a crystal structure of 2-(4-Fluorophenyl)-6-phenyltetrahydro-2*H*-thiopyran-4-one 1-oxide, wherein the thiopyran unit is in chair form. The molecular structure of the title compound, with atomic numbering scheme, is shown in Fig. 1. The thiopyran unit of the title molecule, C_17_H_15_ClO_2_S, is in the chair form. The geometry around S1 atom is tetrahedral and C4 is planar. A crystallographic mirror plane bisects the molecule, passing through the O=S and the opposite C=O atoms of the central ring. The (*p*-chloro)phenyl at the 2 position and the phenyl ring at the 6 position have equatorial orientations. C2—H2···O1(*x*, *y*, 1 + *z*) and C15—H15···Cl1(1/2 - *x*, -*y*, -1/2 + *z*) intermolecular hydrogen bonds forming an infinite one dimensional chain with base vector [0 0 1] are found in the crystal structure. Further, a short intermolecular O1···C4(*x*, *y*, -1 + *z*) contact of 2.970 (5)Å is also found in the crystal structure.

### Experimental

A mixture of *cis*-2-(4-chlorophenyl)-6-phenyldithian-4-one (3.18 g, 0.01 mol), diethyl ether (60 ml), bromine (3.0 g) in water (30 ml) was shaken for few minutes. The solid that separated was filtered, washed with ether and recrystallized from chloroform-carbon tetrachloride mixture (1:1 *v*/*v*). The yield obtained was 68%(2.16 g).

### Refinement

The structure was solved in the space group *Pnma* with half a molecule in the asymmetric unit. The other half is related by a mirror plane symmetry [*x*, 1/2 - *y*, *z*]. The s.o.f. of C14A, Cl1, C14B and H14B is 0.5 and for the remaining phenyl group atoms it is 1.00. This confirms the (*p*-chloro)phenyl group at 2 position and the phenyl group at 6 position. The H atoms were positioned geometrically and allowed to ride on their parent atoms with C—H = 0.93–0.98 Å and *U*_iso_ = 1.2*U*_eq_(C).

### Crystal data

**Table:**

C_17_H_15_ClO_2_S	*D*_x_ = 1.366 Mg m^−^^3^
*M**_r_* = 318.81	Melting point: 417 K
Orthorhombic, *P**n**m**a*	Mo *K*α radiation λ = 0.71073 Å
Hall symbol: -P 2ac 2n	Cell parameters from 9562 reflections
*a* = 11.5195 (5) Å	θ = 3.0–24.8º
*b* = 25.7589 (12) Å	µ = 0.38 mm^−^^1^
*c* = 5.2248 (2) Å	*T* = 296 (2) K
*V* = 1550.35 (12) Å^3^	Thick, colourless
*Z* = 4	0.58 × 0.35 × 0.15 mm
*F*_000_ = 664	

### Data collection

**Table:**

Bruker APEXII CCD diffractometer	1954 independent reflections
Radiation source: fine-focus sealed tube	1551 reflections with *I* > 2σ(*I*)
Monochromator: graphite	*R*_int_ = 0.040
Detector resolution: 0 pixels mm^-1^	θ_max_ = 28.3º
*T* = 296(2) K	θ_min_ = 1.6º
φ and ω scans	*h* = −15→15
Absorption correction: multi-scan(SADABS; Bruker, 2004)	*k* = −34→34
*T*_min_ = 0.809, *T*_max_ = 0.945	*l* = −6→6
33450 measured reflections	

### Refinement

**Table:**

Refinement on *F*^2^	Secondary atom site location: difference Fourier map
Least-squares matrix: full	Hydrogen site location: inferred from neighbouring sites
*R*[*F*^2^ > 2σ(*F*^2^)] = 0.061	H-atom parameters constrained
*wR*(*F*^2^) = 0.211	*w* = 1/[σ^2^(*F*_o_^2^) + (0.0825*P*)^2^ + 1.5305*P*] where *P* = (*F*_o_^2^ + 2*F*_c_^2^)/3
*S* = 1.20	(Δ/σ)_max_ = 0.001
1954 reflections	Δρ_max_ = 0.32 e Å^−^^3^
106 parameters	Δρ_min_ = −0.42 e Å^−^^3^
Primary atom site location: structure-invariant direct methods	Extinction correction: none

### Special details

**Table:**

Geometry. Bond distances, angles *etc*. have been calculated using the rounded fractional coordinates. All su's are estimated from the variances of the (full) variance-covariance matrix. The cell e.s.d.'s are taken into account in the estimation of distances, angles and torsion angles
Refinement. Refinement of *F*^2^ against ALL reflections. The weighted *R*-factor *wR* and goodness of fit *S* are based on *F*^2^, conventional *R*-factors *R* are based on *F*, with *F* set to zero for negative *F*^2^. The threshold expression of *F*^2^ > 2σ(*F*^2^) is used only for calculating *R*-factors(gt) *etc*. and is not relevant to the choice of reflections for refinement. *R*-factors based on *F*^2^ are statistically about twice as large as those based on *F*, and *R*- factors based on ALL data will be even larger.

### Fractional atomic coordinates and isotropic or equivalent isotropic displacement parameters (Å^2^)

**Table:**

	*x*	*y*	*z*	*U*_iso_*/*U*_eq_	Occ. (<1)
Cl1	0.0955 (4)	0.00160 (11)	0.0431 (9)	0.1475 (18)	0.500
S1	0.24636 (8)	0.25000	0.27287 (17)	0.0434 (3)	
O1	0.3171 (3)	0.25000	0.0330 (5)	0.0614 (10)	
O4	0.5597 (3)	0.25000	0.7542 (7)	0.0772 (13)	
C2	0.3029 (3)	0.19728 (12)	0.4726 (5)	0.0477 (9)	
C3	0.4354 (3)	0.20065 (15)	0.4859 (7)	0.0621 (13)	
C4	0.4819 (3)	0.25000	0.5959 (8)	0.0581 (16)	
C11	0.2583 (3)	0.14695 (14)	0.3651 (7)	0.0593 (11)	
C12	0.1618 (4)	0.12411 (19)	0.4657 (10)	0.0873 (18)	
C13	0.1167 (5)	0.0784 (2)	0.3692 (16)	0.113 (3)	
C14A	0.1685 (7)	0.0557 (2)	0.1666 (16)	0.112 (3)	0.500
C14B	0.1685 (7)	0.0557 (2)	0.1666 (16)	0.112 (3)	0.500
C15	0.2634 (7)	0.0778 (3)	0.0566 (14)	0.124 (3)	
C16	0.3087 (5)	0.1234 (2)	0.1524 (10)	0.0940 (19)	
H2	0.27192	0.20147	0.64603	0.0573*	
H3A	0.46377	0.17188	0.58808	0.0745*	
H3B	0.46629	0.19651	0.31439	0.0745*	
H12	0.12502	0.13981	0.60394	0.1053*	
H13	0.05139	0.06340	0.44321	0.1358*	
H14B	0.13910	0.02476	0.10157	0.1344*	0.500
H15	0.29807	0.06199	−0.08411	0.1492*	
H16	0.37304	0.13854	0.07466	0.1130*	

### Atomic displacement parameters (Å^2^)

**Table:**

	*U*^11^	*U*^22^	*U*^33^	*U*^12^	*U*^13^	*U*^23^
Cl1	0.168 (3)	0.0895 (18)	0.185 (4)	−0.0163 (19)	−0.060 (3)	−0.035 (2)
S1	0.0328 (5)	0.0708 (7)	0.0265 (5)	0.0000	−0.0033 (3)	0.0000
O1	0.0611 (19)	0.101 (2)	0.0221 (13)	0.0000	0.0046 (13)	0.0000
O4	0.0365 (16)	0.138 (3)	0.057 (2)	0.0000	−0.0168 (14)	0.0000
C2	0.0413 (14)	0.0715 (18)	0.0304 (13)	0.0054 (13)	−0.0019 (11)	0.0014 (12)
C3	0.0390 (15)	0.096 (3)	0.0512 (18)	0.0139 (16)	−0.0103 (13)	−0.0018 (17)
C4	0.0304 (19)	0.106 (4)	0.038 (2)	0.0000	−0.0045 (16)	0.0000
C11	0.0562 (18)	0.069 (2)	0.0527 (18)	0.0096 (16)	−0.0125 (15)	0.0000 (15)
C12	0.063 (2)	0.095 (3)	0.104 (4)	−0.007 (2)	0.009 (2)	−0.018 (3)
C13	0.092 (4)	0.097 (4)	0.150 (6)	−0.019 (3)	−0.005 (4)	−0.004 (4)
C14A	0.118 (5)	0.080 (3)	0.138 (5)	−0.001 (3)	−0.048 (4)	−0.017 (3)
C14B	0.118 (5)	0.080 (3)	0.138 (5)	−0.001 (3)	−0.048 (4)	−0.017 (3)
C15	0.156 (6)	0.099 (4)	0.118 (5)	0.008 (4)	−0.006 (4)	−0.045 (4)
C16	0.111 (4)	0.094 (3)	0.077 (3)	−0.001 (3)	0.013 (3)	−0.025 (3)

### Geometric parameters (Å, °)

**Table:**

Cl1—C14A	1.751 (7)	C13—C14B	1.349 (11)
Cl1—C14B	1.751 (7)	C14A—C15	1.360 (11)
Cl1—H14B	0.8400	C14B—C15	1.360 (11)
S1—C2	1.832 (3)	C15—C16	1.379 (9)
S1—C2^i^	1.832 (3)	C2—H2	0.9800
S1—O1	1.495 (3)	C3—H3A	0.9700
O4—C4	1.220 (5)	C3—H3B	0.9700
C2—C3	1.530 (5)	C12—H12	0.9300
C2—C11	1.503 (5)	C13—H13	0.9300
C3—C4	1.494 (4)	C14A—H14B	0.9300
C11—C12	1.363 (6)	C14B—H14B	0.9300
C11—C16	1.393 (6)	C15—H15	0.9300
C12—C13	1.382 (8)	C16—H16	0.9300
C13—C14A	1.349 (11)		
			
Cl1···C15^ii^	3.645 (9)	C2···O1^xiv^	3.232 (4)
Cl1···H15^iii^	2.8200	C2···O1^x^	3.232 (4)
S1···O4^iv^	3.494 (4)	C3···O1^xiv^	3.412 (4)
S1···O4^v^	3.276 (4)	C3···O1^x^	3.412 (4)
S1···C4^iv^	3.605 (4)	C4···S1^xii^	3.605 (4)
S1···O4^vi^	3.494 (4)	C4···O1^xiv^	2.970 (5)
S1···O4^vii^	3.276 (4)	C4···S1^xv^	3.605 (4)
S1···C4^vi^	3.605 (4)	C4···O1^x^	2.970 (5)
O1···O4^viii^	3.152 (5)	C12···C15^xiv^	3.511 (9)
O1···C2^viii^	3.232 (4)	C15···C12^viii^	3.511 (9)
O1···C3^viii^	3.412 (4)	C15···Cl1^iii^	3.645 (9)
O1···C4^viii^	2.970 (5)	C16···O1	3.322 (5)
O1···C16	3.322 (5)	C3···H16	2.7700
O1···C2^ix^	3.232 (4)	C16···H3B	2.7500
O1···C3^ix^	3.412 (4)	H2···O1^xiv^	2.4300
O1···O4^ix^	3.152 (5)	H2···H12	2.3300
O1···C16^i^	3.322 (5)	H2···O4^v^	2.7900
O1···C4^ix^	2.970 (5)	H2···O4^vii^	2.7900
O4···O1^x^	3.152 (5)	H2···O1^x^	2.4300
O4···S1^xi^	3.276 (4)	H2···H2^i^	2.5000
O4···S1^xii^	3.494 (4)	H3A···H12^xi^	2.5900
O4···S1^xiii^	3.276 (4)	H3B···O1	2.6500
O4···O1^xiv^	3.152 (5)	H3B···C16	2.7500
O4···S1^xv^	3.494 (4)	H3B···H16	2.2300
O1···H3B^i^	2.6500	H12···H2	2.3300
O1···H2^viii^	2.4300	H12···H3A^vii^	2.5900
O1···H2^ix^	2.4300	H15···Cl1^ii^	2.8200
O1···H3B	2.6500	H16···C3	2.7700
O4···H2^xiii^	2.7900	H16···H3B	2.2300
O4···H2^xi^	2.7900		
			
C14A—Cl1—H14B	8.00	C11—C16—C15	120.2 (5)
C14B—Cl1—H14B	8.00	S1—C2—H2	108.00
C2—S1—C2^i^	95.66 (14)	C3—C2—H2	108.00
O1—S1—C2	106.48 (13)	C11—C2—H2	108.00
O1—S1—C2^i^	106.48 (13)	C2—C3—H3A	109.00
C3—C2—C11	114.0 (3)	C2—C3—H3B	109.00
S1—C2—C3	109.8 (2)	C4—C3—H3A	109.00
S1—C2—C11	107.8 (2)	C4—C3—H3B	108.00
C2—C3—C4	115.0 (3)	H3A—C3—H3B	108.00
C3—C4—C3^i^	116.6 (3)	C11—C12—H12	119.00
O4—C4—C3	121.62 (19)	C13—C12—H12	119.00
O4—C4—C3^i^	121.62 (19)	C12—C13—H13	120.00
C12—C11—C16	117.4 (4)	C14A—C13—H13	120.00
C2—C11—C16	122.1 (4)	C14B—C13—H13	120.00
C2—C11—C12	120.5 (3)	Cl1—C14A—H14B	8.00
C11—C12—C13	122.2 (5)	C13—C14A—H14B	120.00
C12—C13—C14A	119.3 (6)	C15—C14A—H14B	120.00
C12—C13—C14B	119.3 (6)	Cl1—C14B—H14B	8.00
Cl1—C14A—C15	124.3 (6)	C13—C14B—H14B	120.00
Cl1—C14A—C13	115.0 (6)	C15—C14B—H14B	120.00
C13—C14A—C15	120.4 (6)	C14A—C15—H15	120.00
Cl1—C14B—C15	124.3 (6)	C14B—C15—H15	120.00
C13—C14B—C15	120.4 (6)	C16—C15—H15	120.00
Cl1—C14B—C13	115.0 (6)	C11—C16—H16	120.00
C14A—C15—C16	120.5 (7)	C15—C16—H16	120.00
C14B—C15—C16	120.5 (7)		
			
O1—S1—C2—C3	48.2 (2)	C2—C11—C16—C15	−178.6 (5)
O1—S1—C2—C11	−76.5 (2)	C12—C11—C16—C15	−2.2 (7)
C2^i^—S1—C2—C3	−60.9 (2)	C11—C12—C13—C14A	−0.9 (9)
C2^i^—S1—C2—C11	174.5 (2)	C11—C12—C13—C14B	−0.9 (9)
S1—C2—C3—C4	59.9 (3)	C12—C13—C14A—Cl1	−174.1 (5)
C11—C2—C3—C4	−179.1 (3)	C12—C13—C14A—C15	−0.7 (11)
S1—C2—C11—C12	−96.7 (4)	C12—C13—C14B—Cl1	−174.1 (5)
S1—C2—C11—C16	79.6 (4)	C12—C13—C14B—C15	−0.7 (11)
C3—C2—C11—C12	141.3 (4)	Cl1—C14A—C15—C16	173.5 (6)
C3—C2—C11—C16	−42.5 (5)	C13—C14A—C15—C16	0.7 (11)
C2—C3—C4—O4	134.6 (4)	Cl1—C14B—C15—C16	173.5 (6)
C2—C3—C4—C3^i^	−50.5 (4)	C13—C14B—C15—C16	0.7 (11)
C2—C11—C12—C13	178.7 (5)	C14A—C15—C16—C11	0.8 (10)
C16—C11—C12—C13	2.3 (7)	C14B—C15—C16—C11	0.8 (10)

Symmetry codes: (i) *x*, −*y*+1/2, *z*; (ii) −*x*+1/2, −*y*, *z*−1/2; (iii) −*x*+1/2, −*y*, *z*+1/2; (iv) *x*−1/2, −*y*+1/2, −*z*+1/2; (v) *x*−1/2, −*y*+1/2, −*z*+3/2; (vi) *x*−1/2, *y*, −*z*+1/2; (vii) *x*−1/2, *y*, −*z*+3/2; (viii) *x*, *y*, *z*−1; (ix) *x*, −*y*+1/2, *z*−1; (x) *x*, −*y*+1/2, *z*+1; (xi) *x*+1/2, *y*, −*z*+3/2; (xii) *x*+1/2, −*y*+1/2, −*z*+1/2; (xiii) *x*+1/2, −*y*+1/2, −*z*+3/2; (xiv) *x*, *y*, *z*+1; (xv) *x*+1/2, *y*, −*z*+1/2.

### Hydrogen-bond geometry (Å, °)

**Table:**

*D*—H···*A*	*D*—H	H···*A*	*D*···*A*	*D*—H···*A*
C2—H2···O1^xiv^	0.98	2.43	3.232 (4)	138
C15—H15···Cl1^ii^	0.93	2.82	3.745 (9)	170

Symmetry codes: (xiv) *x*, *y*, *z*+1; (ii) −*x*+1/2, −*y*, *z*−1/2.
